# Tests for Early Diagnosis of Cardiovascular Autonomic Neuropathy: Critical Analysis and Relevance

**DOI:** 10.3389/fendo.2013.00173

**Published:** 2013-11-11

**Authors:** Luiz Clemente Rolim, José Sérgio Tomaz de Souza, Sérgio Atala Dib

**Affiliations:** ^1^Department of Internal Medicine, Discipline of Endocrinology, Diabetes Center, Universidade Federal de São Paulo, São Paulo, Brazil; ^2^Department of Neuropsychiatry and Behavioral Science, Universidade Federal de Pernambuco, Recife, Brazil

**Keywords:** diabetic complications, diabetic autonomic neuropathy, early diagnosis, heart rate variability, cardiac function tests

## Initial Considerations

Despite its high prevalence in individuals with diabetes mellitus (DM) neuropathies are the most underdiagnosed and undertreated diabetic chronic complication (1). The involvements of somatic and autonomic nerve fibers in DM present complex pathophysiologies (1–4). The impairment of sympathetic and parasympathetic divisions of the autonomic nervous system (ANS) leads to diabetic autonomic neuropathy (DAN), a condition that may affect different organ systems such as cardiovascular, gastrointestinal, genitourinary, sudomotor, and visual (4). Cardiovascular autonomic neuropathy (CAN), within the context of DAN, occurs when there is an impairment of autonomic control of the cardiovascular system after ruling out other causes of dysautonomia (1).

It is known that CAN is an early and frequent complication of DM, affecting from 7 to 15% of newly diagnosed patients to 90% of those in line for a double transplant. In addition, CAN is among one of the most disabling complications of DM in terms of life expectancy and quality.

Clinical manifestations of CAN are pleomorphic and appear in late stages, and in isolation do not present enough sensitivity and specificity for diagnosis requiring the use of objective autonomic tests (3, 4). Thus, detection of CAN in a diabetic patient requires sensitive and specific tests in order to establish differential diagnosis and quantify the severity of dysautonomia (3). Specifically, the presence of symptoms or signs suggestive of autonomic changes – such as erectile dysfunction, dizziness, intermittent visual impairment, postprandial hypotension, resting tachycardia, or exercise intolerance (dyspnea) in persons with DM – should be investigated and confirmed by performing objective diagnostic tests for CAN (3, 4).

## Diagnosis of Cardiovascular Autonomic Neuropathy

The recent Toronto Consensus concluded that currently the five most sensitive and specific methods to assess the presence of CAN are (4): (A) Study of heart rate variability (HRV) using the ratio of the RR intervals of the electrocardiogram (ECG); (B) Baroreflex sensitivity (BRS); (C) Muscle sympathetic nerve activity (MSNA); (D) Measurement of plasma levels of catecholamines (PLC); (E) Cardiac sympathetic mapping (CSM).

Heart rate (HR) variability is recognized as a chaotic signal with hidden constituents. HRV can be defined as an oscillation of RR intervals between each heart beat that occurs as a result of ANS sympathetic and parasympathetic activities on the sinus node (6, 7). The hypothesis that reduction of HRV reflects the suppression of vagal modulation and sympathetic dominance, resulting in higher mortality and arrhythmia, has been used as the basis for numerous studies, which have consistently confirmed this relationship (2, 4, 5, 7).

Heart rate variability measurements are obtained with an analysis of spontaneous or experimentally induced fluctuations of RR intervals in the ECG. The methods currently accepted include cardiovascular autonomic reflex tests (CARTs) (experimentally induced variations of RR) and time and frequency-domain methods (spontaneous variations of RR). Time and frequency-domain tests measure, respectively, the overall magnitude of the fluctuations of RR intervals between each heart beat around the average value and the magnitude of fluctuations in a predetermined range of frequency. Time-domain measurements can be assessed by statistical analysis of RR intervals while frequency-domain measurements by spectral analysis of an array (1–4, 6–8).

Spectral analysis uses a mathematical algorithm (autoregression analysis or fast Fourier transform) to turn HRV – a complex biological signal – into its causal components, presenting them according to the frequency in which they alter the RR (1, 2, 6, 7). The result (spectral amplitude) is presented in a graph consisting of Amplitude (*Y* axis) vs. Frequency (*X* axis). The spectral amplitude does not only reflect the magnitude of HRV (*Y* axis), but also the oscillations in different frequencies, i.e., the number of HR fluctuations per second (*X* axis). It has been demonstrated that the total spectral amplitude (total power or TP) of HRV consists of three key frequency bands:

(1) Very low-frequency component or VLF (from 0.01 to 0.04 Hz): this component is related to the fluctuations in vasomotor tonus associated with thermoregulation and sweating (sympathetic control);
(2) Low-frequency component or LF (from 0.04 to 0.15 Hz): this component is related to the baroreflex (sympathetic control with vagal modulation);
(3) High-frequency component or HF (from 0.15 to 0.5 Hz): this component is related to changes in RR according to the phases of breathing (inhale/exhale), which are under parasympathetic control.

In spite of ambulatory blood pressure monitoring (ABPM) being a reliable tool to assess BP patterns within 24 h, ABPM as well as the QT interval (QTi) are tests that are not sensitive enough for the diagnosis of CAN (3, 4), however, when such tests are altered CARTs should be performed (level B recommendation).

Baroreflex sensitivity is a technique used to assess cardiac baroreflex function by combining information from HR and blood pressure (BP). Theoretically, this technique evaluates the two afferent sections of the cardiovascular ANS: sympathetic (arterial vasoconstriction) and vagal (bradycardia or tachycardia) in response to changes induced in BP. In practice, however, only the cardio-vagal section ends up being analyzed due to technical difficulties in assessing arteriolar sympathetic tone. To date, there is no data on the sensitivity and specificity of this method for diagnosing CAN, and the technique requires continuous monitoring of BP through *finapress*.

Muscle sympathetic nerve activity is an invasive method (and not feasible in routine clinical practice) to directly assess conduction of the peripheral sympathetic nervous system through microneurography. Routinely, it is not recommended for the diagnosis of CAN.

Evaluating the PLC (adrenaline, noradrenaline, and its metabolites) has not been proven to be useful for the staging or diagnosis of CAN, although PLC has a remarkable role in the differential diagnosis of other endocrine pathologies such as pheochromocytoma and medullary adrenal insufficiency.

Cardiac sympathetic imaging techniques (PET and SPECT) directly assess sympathetic innervation through scintigraphy or CSM using radiolabeled catecholamines actively captured by sympathetic nerve terminals. Due to its high cost, lacks of reference values and of standardized methodology, and also its susceptibility to ischemia interference (because the result depends directly on myocardial perfusion), these techniques are not recommended for routine diagnosis of CAN and currently remain restricted to the research field.

In summary, early diagnosis of CAN is imperative in patients with DM, both type 2 (from diagnosis) and type 1 (5 years after diagnosis). Currently, CARTs are the gold standard for diagnosing CAN in persons with DM (6, 8) and include four tests: (i) deep breathing (E:I), (ii) Valsalva, (iii) orthostatic (30:15), and (iv) orthostatic hypotension (OH). Maneuvers used in the first three tests induce changes in HRV that primarily assess the parasympathetic ANS [level B recommendation by the Italian (3) and Toronto (4) Consensuses]. In contrast, OH or the variation in systolic BP in supine and standing positions evaluates the function of the sympathetic ANS (level B recommendation) (3, 4). A review of the technical aspects of the methodology used by Diabetic Neuropathy Study Group of the Italian Society of Diabetology can be found in reference (3) while the protocol used by the authors at the Federal University of São Paulo (Diabetes Center) can be found in reference (2).

## Critical Analysis of HRV

In 1996, a task force directed by the European Society of Cardiology and the American Society of Electrophysiology proposed the standardization of parameters and the clinical use of autonomic tests for the diagnosis of CAN, specifically in DM (6). The proposed guidelines met the objectives, however, they emphasized computational measurement and techniques to the detriment of the physiological interpretations of HRV. Furthermore, consistent values for normal measurements of HRV in different populations and by age bracket and gender were not defined (6, 10).

Technological progress, through high level computerization, has allowed a broad application of methods to study the ANS through HRV analysis. In these methods, the biological signs of the ANS are obtained indirectly and may be affected by factors unrelated to the activity of the ANS, thereby leading to potentially inadequate or confusing results. We emphasize the need for knowledge of physiology and pathophysiology in order to correctly understand, apply, and interpret autonomic tests, as well as the various factors that can alter their results, such as age, gender, body position, time of day, prior physical activity, nutrition, BP, HR, respiratory rate baseline, coffee ingestion, use of cigarettes, mental stress, hyperglycemia and hypoglycemia, and insulinemia. Hyperinsulinemia seems to be related to an increase in plasma norepinephrine and a decrease in parasympathetic control of HR caused by insulin itself (11). Furthermore, insulin affects blood vessel tone and can induce vasodilation and hypotension. Besides the above named factors that may influence the outcome of autonomic tests, it is crucial to reinforce that the biological signal (HRV) has to be obtained through the ECG (RR intervals), and that a capable professional must dominate its interpretation because a single unrecognized extrasystole could distort all test results (6, 10).

Recent efforts, through consensus of authorities, recommend the adoption of standardized techniques in the application and interpretation of autonomic tests according to the pathophysiology and confounding factors present in cardiovascular examinations. The Toronto (4) and Italian (3) Consensuses treated CARTs as the gold standard for the diagnosis of CAN in DM.

The Italian Consensus (3) established the lack of an individual and effective test for the diagnosis of CAN. In contrast, the application of various tests (which assess both divisions of the ANS, parasympathetic, and sympathetic) reduces the likelihood of false positives (3, 4, 8). The Italian Consensus (3) omitted the other ways of measuring HRV, however, the international Toronto Consensus (4, 8) advocates the use of a set of HRV measurements beyond the four reflex tests currently considered the gold standard. For example, adding three tests of analysis in the frequency-domain (spectral analysis) to the CARTs (four tests), totaling seven tests (1, 2, 4, 8).

## Clinical Relevance

Cardiovascular autonomic neuropathy has been recognized as a significant cause of morbidity and mortality in diabetic patients since the 1970s. But only in recent years CAN was proven to have a predictive power for primary cardiovascular events (non-fatal myocardial infarction, stroke, and sudden cardiac death) greater than the classical risk factors such as smoking, LDL levels, and family history of coronary artery disease. DIAD-1 (12) and DIAD-3 (13) prospective studies showed unequivocally that the abnormal Valsalva test resulted in a relative risk 3.0 and 4.5 times higher for primary cardiovascular event and silent myocardial ischemia, respectively (5, 12, 13). Further reinforcing such concepts, one of the greatest legacies of the well-known ACCORD study was precisely the role of cardiovascular dysautonomia, which doubled the risk of death in diabetics with CAN and concomitant sensory-motor polyneuropathy (14).

Recently, the Toronto Consensus established four reasons why the diagnosis of CAN is relevant to clinical practice:

(1) For diagnosing and staging the different clinical forms of CAN: initial, definite, and advanced or severe;
(2) For the differential diagnosis of clinical manifestations (e.g., resting tachycardia, OH, and dyspnea upon exercise) and their respective treatment;
(3) For stratifying the degree of cardiovascular risk and the risk of other diabetic complications (nephropathy, retinopathy, and silent myocardial ischemia);
(4) To adapt the goal of glycated hemoglobin (HbA1c) in each patient: for example, those with severe CAN should have a less aggressive glycemic control due to the risk of asymptomatic hypoglycemia in these patients while patients with initial stages of CAN should have a more intensive glycemic control.

The main clinical indications of the autonomic reflex tests are summarized in Table 1.

**Table 1:** Indications for the cardiovascular autonomic reflex tests (CARTs)*.

**1**.	Diagnosis and Staging of CAN in Type 2 DM patients (at diagnosis and annually thereafter)
**2**.	Diagnosis and Staging of CAN in Type 1 DM patients (5 years after diagnosis and annually thereafter)
**3**.	Stratification of Cardiovascular risk: in pre-operatory testing, pre-physical activity, indication of selective beta-blocker, and suspected silent ischemia
**4**.	Differential Diagnosis of other manifestations of DAN (regardless of DM duration): assess whether gastroparesis, erectile dysfunction, orthostatic hypotension, dizziness, syncope, or tachycardia in diabetic persons are due to dysautonomia
**5**.	Evaluate the progression of autonomic failure and monitor response to therapy (e.g., continuous infusion of insulin, post-transplants, and use of antioxidants)
**6**.	Differential diagnosis of other causes of neuropathy such as autoimmune autonomic neuropathy (CIDP, Celiac Disease, Amyotrophy) or toxic-infectious neuropathy (alcohol, Hansen, HIV) as well as in cases where the presence of autonomic neuropathy is disproportionate to the sensory-motor neuropathy

*Modified from Ref. (3) and (4).

DM-2, Type 2 diabetes mellitus; DM-1, Type 1 diabetes mellitus; DAN, diabetic autonomic neuropathy; CIDP, chronic inflammatory demyelinating polyneuropathy; HIV, human immunodeficiency virus.

Although it goes beyond the scope of this analysis, it is worth remembering that two lines of research have shown promising results in the treatment of diabetic CAN: (1) A still-experimental line in rats using “chaperones” (heat shock proteins) (15), and (2) A phase three of human therapeutic clinical trial utilizing weekly subcutaneous C-peptide in patients with type 1 DM and polyneuropathy with dysautonomia (16).

## Final Considerations

There is an apparent neglect in the diagnosis of CAN in diabetic persons as result of: low interest in an unfamiliar complication, skepticism concerning therapeutic options, lack of understanding diagnostic utility, and the necessity of education and training related to cardiovascular tests, as pointed out by the Italian Consensus (3), in spite of increasingly consistent evidence of its predictive value for cardiovascular morbidity and mortality (14).

Despite the Toronto Consensus (4) having determined HRV analysis to be the most sensitive and specific method, there is no unanimous criteria for the diagnosis of CAN (4, 8). Furthermore, there is controversy regarding the best way to include measurements of the ANS in the daily clinical routine (9). In a recent review, Vinik (7) affirms emphatically the need to promote more aggressive strategies and therapeutic approaches in favor of patients with CAN. A proactive position toward diabetics with burning feet or any suspected dysautonomia requires an objective, safe and accurate investigation of CAN through the use of CARTs.

In conclusion, CARTs as well as time and frequency-domain HRV analysis provide key information regarding the sympathetic and parasympathetic modulation of the cardiovascular system; all of which represent a clinically relevant method for the diagnosis of CAN. However, the correct application of the technique (RR intervals of the ECG) is critical, and the methodology depends, also critically, on the correct understanding of the underlying physiological and pathophysiological mechanisms, the mathematical model used, the bias factors, and possible technical artifacts.

## Authors Contribution

Luiz Clemente Rolim and José Sérgio Tomaz de Souza search the literature and write the manuscript. SAD contributed to manuscript preparation. All authors read and approved the final manuscript.
